# Pixel-by-pixel precise delay correction for measurement of cerebral hemodynamic parameters in H_2_^15^O PET study

**DOI:** 10.1007/s12149-017-1156-5

**Published:** 2017-02-27

**Authors:** Muhammad M. Islam, Tetsuya Tsujikawa, Tetsuya Mori, Yasushi Kiyono, Hidehiko Okazawa

**Affiliations:** Biomedical Imaging Research Center, University of Fukui, 23-3, Matsuoka-Shimaizuki, Eiheiji-cho, Fukui, 910-1193 Japan

**Keywords:** H_2_^15^O PET, Delay correction, Pixelwise calculation, Cerebral blood flow, Arterial-to-capillary blood volume (*V*_0_)

## Abstract

**Objective:**

A new method of delay time estimation was proposed to measure precise cerebral blood flow (CBF) and arterial-to-capillary blood volume (*V*_0_) using ^15^O-water PET.

**Methods:**

Nineteen patients with unilateral arterial stenoocclusive lesions were studied to evaluate hemodynamic status before treatment. The delay time of each pixel was calculated using least squares fitting with an arterial blood input curve adjusted to the internal carotid artery counts at the skull base. Pixel-by-pixel delay estimation provided a delay map image that could be used for precise calculation of CBF and *V*_0_ using a one-tissue compartment model, and the values from this method were compared with those from the slice-by-slice correction method.

**Results:**

The affected side showed a longer delay time than the contralateral cerebral hemisphere. Although the mean cortical CBF values were not different between the two methods, the slice-by-slice delay correction overestimated CBF in the hypo perfused area. The scatter plot of *V*_0_ pixel values showed significant difference between the two correction methods where the slice-by-slice delay correction significantly overestimated *V*_0_ in the whole brain (*P* < 0.05).

**Conclusion:**

Pixel-by-pixel delay correction provides delay images as well as better estimation of CBF and *V*_0_, thus offering useful and beneficial information for the treatment of cerebrovascular disease.

## Introduction

Precise measurements of cerebral blood flow (CBF) and other hemodynamic parameters are important in the diagnosis of impaired status in cerebrovascular diseases (CVD). For the quantitative measurement of these parameters using positron emission tomography (PET) and ^15^O-labeled tracers, the steady-state and autoradiographic (ARG) methods based on a single compartment model were proposed to evaluate cerebral hemodynamics [1–4]. A one-tissue compartment model (1-TCM) was applied as the next step, to obtain more precise values of CBF using a better kinetic description for avoiding effects of radioactivity in the vessels and the distribution of water into brain tissues [5–7].

Quantitative values of hemodynamic parameters in the ^15^O-PET with arterial blood sampling are affected by the accuracy of an input function corrected for parameters such as tracer delay time and dispersion constants [4, 8]. The precision of CBF and arterial-to-capillary blood volume (*V*_0_) calculation with a 1-TCM was reported to be sensitive to the delay correction, i.e., the difference between tracer arrival times at brain tissue and the arterial sampling site [6, 9]. To simplify the model parameter estimation and calculate efficiently using the 1-TCM, programs employed a fixed dispersion constant and a single delay time for the whole brain, or slice-by-slice delay correction was applied [6, 7, 9]. However, correction parameters for the whole brain or for each brain slice may cause errors in calculation of hemodynamic parameters because the values could be affected by estimation errors of delay and dispersion of the arterial input.

Delay time estimation is not so simple because it varies according to the brain structure and arterial distribution or collaterals that supply regional blood flow [10–12]. Furthermore, since the brain is a heterogeneous mixture of gray and white matter with different amounts of blood flow, each voxel in the brain requires a unique delay and dispersion correction for ^15^O-tracer arrival even within the same slice [2, 4, 13]. Patients with unilateral arterial stenoocclusive lesions would have different delays in each hemisphere [14, 15]; however, slice-by-slice adjustment of the delay correction applies a single input function corrected for delay and dispersion time to all pixels in the same slice level despite different tracer arrival times for each pixel in the slice, which may not reflect appropriate CBF and *V*_0_ values over the entire brain because of substantial biases in regional delay estimation [14].

This study was designed to evaluate the effects of delay time correction by pixel-by-pixel estimation for precise measurements of hemodynamic parameters such as CBF and *V*_0_ with a 1-TCM [6, 7]. The CBF and *V*_0_ values from this new correction method were compared with those obtained from the standard method using slice-by-slice delay correction [7, 11].

## Materials and methods

### Subjects

Nineteen patients (18 males and 1 female, mean age = 68.8 ± 9.8 y.o.) with unilateral stenoocclusive disease in the major cerebral arteries were included in the study. Twelve patients had stenosis and five patients had occlusions in the unilateral internal carotid artery (ICA), one had a middle cerebral artery (MCA) occlusion, and the other had a common carotid artery occlusion. Ten patients had transient or minor persistent symptoms of transient ischemic attack (TIA), probably due to stenoocclusive CVD, and seven of them had lacunar infarctions on MRI. The other nine patients were asymptomatic. They underwent a ^15^O-PET study to evaluate their hemodynamic status before deciding on further treatment because the Japanese Guidelines for the Management of Stroke published by the Japan Stroke Society recommends PET or SPECT studies to detect regions of severely impaired cerebral hemodynamics before extracranial–intracranial (EC–IC) bypass surgery or other neurosurgical treatments. The PET study was approved by the Ethics Committee of our institute, and written informed consent was obtained from each patient. This study was designed retrospectively to improve accuracy of hemodynamic PET parameters using the ^15^O-PET data.

### PET data acquisition

A whole-body PET scanner (Advance, GE Medical Systems, Milwaukee, WI, USA) was used for PET data acquisition. The scanner permits simultaneous acquisition of 35 image slices in 2-dimensional mode with an interslice spacing of 4.25 mm [16]. Performance tests showed the intrinsic resolution of the scanner to be 4.6–5.7 mm in the transaxial direction and 4.0–5.3 mm in the axial direction. The PET data were reconstructed using a Hanning filter with a resolution of 5.0 mm full-width at half-maximum in the transaxial direction and a 128 × 128 matrix in 2 × 2 × 4.25 mm voxel size. Patients were positioned on the scanner bed with their heads immobilized using a head holder. A small cannula was placed in the right brachial artery for blood sampling. A 10-min transmission scan was acquired before the emission scan with a ^68^Ge/^68^Ga rod source for attenuation correction. ^15^O-water and ^15^O-gas (^15^O_2_ and C^15^O) PET studies were performed with arterial blood sampling. Details of the protocol have been described previously [17, 18]. In brief, each subject first inhaled a single dose of C^15^O (1000 MBq) to obtain a cerebral blood volume (CBV) image (mL/100 g). Arterial blood was sampled twice during a 3-min static C^15^O-PET scan started about 50 s after the slow inhalation of C^15^O. Ten minutes after the C^15^O scan, a 3-min static PET acquisition was started with a slow bolus inhalation of ^15^O_2_ (1000 MBq) to obtain images of the oxygen extraction fraction (OEF) and cerebral metabolic rate of oxygen (CMRO_2_) (mL/100 g/min) using the ARG method [19].

Finally, two scans of 3-min dynamic PET acquisition with a bolus injection of 555 MBq ^15^O-water were performed to calculate CBF (mL/100 g/min) images before and 10 min after administration of acetazolamide (ACZ) (1.0 g/60 kg BW). Each dynamic scan consisted of 2 s × 30, 10 s × 6, and 20 s × 3 frames. Radioactivity in the arterial blood during ^15^O_2_ and H_2_^15^O scans was counted by an automatic arterial blood sampling system (ABSS), consisting of a positron radioactivity counter (Apollomec Co. Ltd., Kobe, Japan) and a mini-pump (AC-2120, Atto Co., Tokyo, Japan). Arterial blood was sampled and counted continuously at a constant rate of 7 mL/min for the first 2 min using the ABSS, followed by manual sampling of 0.5 mL of blood every 20 s during the remaining scan time. Radioactivity, as counted by the ABSS, was calibrated with the manually sampled blood at 2 min after tracer administration. Decay of the radioactivity from dynamic PET acquisition and blood data was corrected to the starting point of each scan. The arterial input function from ABSS thus obtained was corrected for dispersion of the external tube in the ABSS described previously [20, 21]. Physiological data such as mean blood pressure, arterial pCO_2_, pO_2_ and O_2_c were measured before and after the ACZ injection to confirm stability of each patient’s condition during the scans.

### Correction of input function

**Fig. 1:**
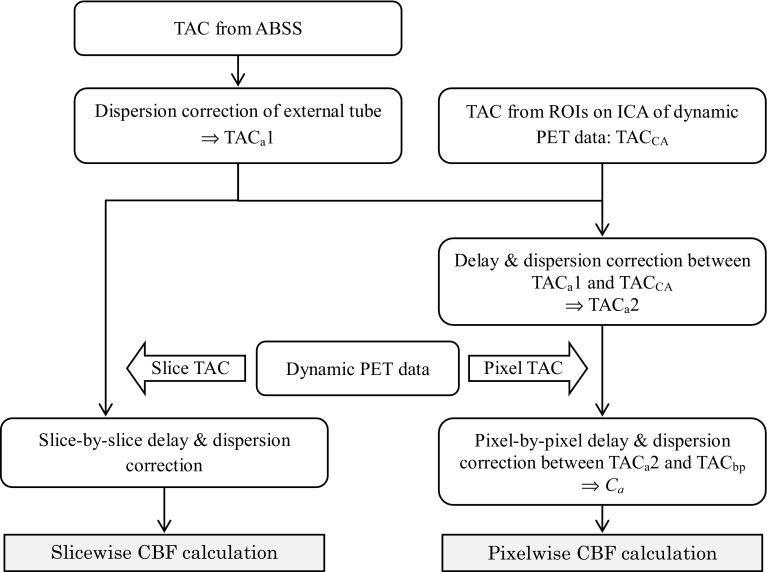
In the slice-by-slice correction method, TAC_a_1 is used for CBF calculation after slicewise delay correction and fixed dispersion (4 s). In the pixel-by-pixel correction method, TAC_a_1 is adjusted to TAC_CA_ (=TAC_a_2) and brain pixel TAC (TAC_bp_) was used for delay and dispersion correction of TAC_a_2. The standard method for delay estimation is slicelwise correction or a single delay correction for the whole brain (*left* flow)

**Fig. 2:**
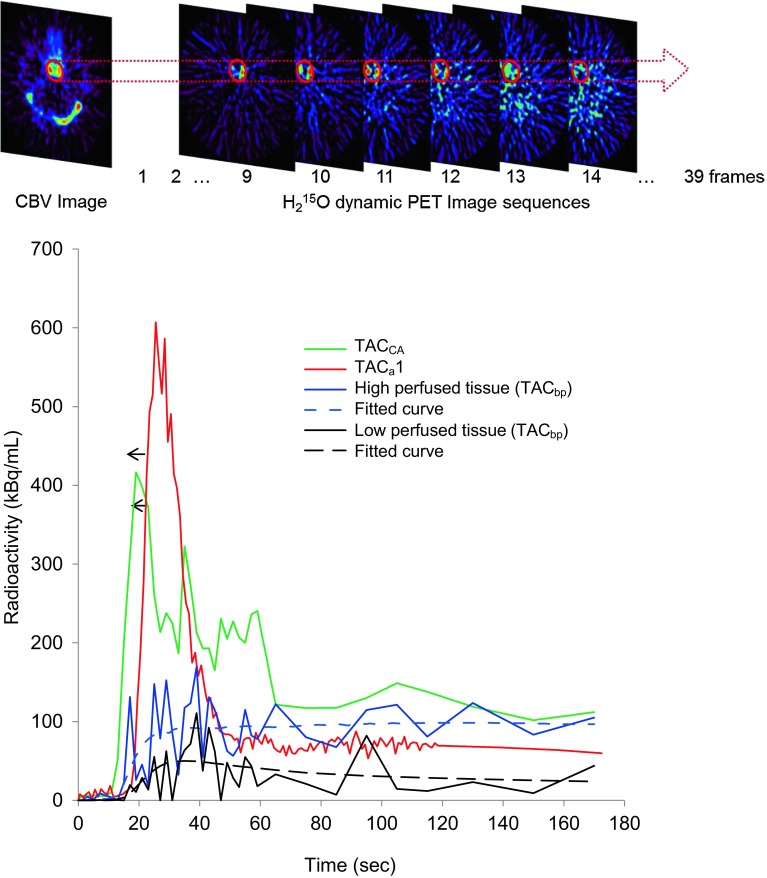
*Upper* Scheme showing the region of interest (ROI) for ICA TAC and its application to the dynamic PET data. *Bottom* Representative TACs from arterial blood sampling (TAC_a_1, *red line*), ROI peak value in the ICA region (TAC_CA_, *green line*) and the regional pixel value in the high and low perfused brain tissue (TAC_bp_, *blue* and *black solid lines*, respectively). *Blue and black dotted lines* (*fitted curve*) show a result of non-linear least *squares* fitting of the arterial input after delay and dispersion correction for each TAC_bp_. The *arrow* shows the direction of delay correction to adjust the initial slope of TAC_a_1 to TAC_CA_. In cases of a long delay time, the difference between TAC_a_1 and TAC_CA_ was greater than 10 s

Dynamic data from each H_2_^15^O PET scan were used for delay correction of the arterial input function at slice-by-slice and pixel-by-pixel level. In the pixel-by-pixel delay correction method, the time difference between the arterial blood sampling point and local brain tissue pixels was estimated in two steps (Fig. 1). First, the time-activity curves (TAC) from arterial blood sampling with external dispersion correction (TAC_a_1) was adjusted to that of the cavernous part of ICA obtained from dynamic PET data (TAC_CA_). The position of the region of interest (ROI) for the TAC_CA_ was determined using the CBV image (Fig. 2). The maximum count in the ROI of each frame was used for the TAC_CA_. The delay time between the TAC_a_1 and TAC_CA_ was estimated using the slope method except for a few cases whose TACs could not be adjusted by the slope method; the least squares fitting approach was applied in these cases [4, 9, 22]. For delay and dispersion correction of the TAC_a_1, the same time constant estimated above was used and the TAC_a_2 was obtained [22, 23].

Second, the delay time of tracer arrival at regional brain tissue from the ICA trunk was estimated for each pixel using the brain pixel TAC (TAC_bp_) and the TAC_a_2. The pixel delay time was determined using least-squares fitting for the initial 60 s of TAC_a_2 and TAC_bp_ with a step size of 0.1 s [9], and a delay image was obtained from the pixelwise delay time estimation. As the delay and dispersion constants are very closely correlated [22, 23], the pixel delay time estimated from TAC_a_2 and TAC_bp_ was also used as the dispersion time constant for the TAC_a_2 correction to obtain the local input function. The local input function (*C*_*a*_), corrected for pixelwise delay and dispersion, was used in the subsequent image calculations.

### Calculation of regional CBF and V_0_

The model for the 1-TCM can be represented by the following equation [6, 7],

1 $$\frac{{{\rm{d}}{{\rm{M}}_{\rm{e}}}(t)}}{{{\rm{d}}t}} = {K_1}{C_{\rm{a}}}(t) - {k_2}{M_{\rm{e}}}(t),$$

where *M*_e_(*t*) (Bq/100 g) is the radioactivity in the brain tissue and *C*_a_(*t*) (Bq/mL) is the true arterial input function. *K*_1_ (mL/100 g/min) and *k*_2_ (1/min) are the rate constants for influx and outflux of the tracer, respectively [7]. *K*_1_ apparently represents regional CBF. The equation is solved as

2 $${M_{\rm{e}}}(t) = {K_1}{C_{\rm{a}}}(t) \otimes {e^{( - {k_2}t)}},$$

where ⊗ represents the convolution. Including the radioactivity in the intravascular spaces, total radioactivity in the brain is expressed as

3 $$M(t) = {K_1}{C_{\text{a}}}(t) \otimes {e^{( - {k_2}t)}} + {V_0}{C_{\text{a}}}(t),$$

where *M*(*t*) (Bq/100 g) is the regional PET count, and *V*_0_ (mL/100 g) is the arterial-to-capillary vascular volume [7]. Here, *K*_1_, *k*_2_ and *V*_0_ can be calculated from measured *M*(t) and *C*_a_(*t*) using the weighted method to reduce the image calculation time [6, 7, 12]. In brief, Eq. 3 can be integrated after weighting with three different weights of *w*_*i*_(*t*) (*i* = 1–3) as below:

4 $$\mathop \smallint \limits_0^T {w_i}\left( t \right)M\left( t \right){\text{d}}t = {K_1}\mathop \smallint \limits_0^T {w_i}\left( t \right){C_{\text{a}}}(t) \otimes {e^{ - {k_2}t}}{\text{d}}t + {V_0}\mathop \smallint \limits_0^T {w_i}\left( t \right){C_{\text{a}}}(t){\text{d}}t,$$

Equation 5 is obtained by rearranging three equations of Eq. 4 to eliminate the *V*_0_ term, where *K*_1_ is cancelled, and various *k*_2_ values can be estimated from the right-hand side ratio using the table look-up method.

5 $$\begin{array}{l} \frac{{\int\limits_0^T {{w_3}(t){C_{\rm{a}}}(t){\rm{d}}t \times } \int\limits_0^T {{w_1}(t)M(t){\rm{d}}t} - \int\limits_0^T {{w_1}(t){C_{\rm{a}}}(t){\rm{d}}t} \times \int\limits_0^T {{w_3}(t)M(t){\rm{d}}t} }}{{\int\limits_0^T {{w_3}(t){C_{\rm{a}}}(t){\rm{d}}t} \times \int\limits_0^T {{w_2}(t)M(t){\rm{d}}t} - \int\limits_0^T {{w_2}(t){C_{\rm{a}}}(t){\rm{d}}t} \times \int\limits_0^T {{w_3}(t)M(t){\rm{d}}t} }}\\ = \frac{{\left[ {\int\limits_0^T {{w_3}(t){C_{\rm{a}}}(t){\rm{d}}t} \times \int\limits_0^T {{w_1}(t){C_{\rm{a}}}(t) \otimes {{\rm{e}}^{{k_2}t}}{\rm{d}}t} - \int\limits_0^T {{w_1}(t){C_{\rm{a}}}(t){\rm{d}}t} \times \int\limits_0^T {{w_3}(t){C_{\rm{a}}}(t) \otimes {{\rm{e}}^{{k_2}t}}{\rm{d}}t} } \right]}}{{{K_1}\left[ {\int\limits_0^T {{w_3}(t){C_{\rm{a}}}(t){\rm{d}}t} \times \int\limits_0^T {{w_2}(t){C_{\rm{a}}}(t) \otimes {{\rm{e}}^{{k_2}t}}{\rm{d}}t} - \int\limits_0^T {{w_2}(t){C_{\rm{a}}}(t){\rm{d}}t} \times \int\limits_0^T {{w_3}(t){C_{\rm{a}}}(t) \otimes {{\rm{e}}^{{k_2}t}}{\rm{d}}t} } \right]}}. \end{array}$$

The *K*_1_ values can be estimated either from the numerator or denominator by substituting the *k*_2_ values in Eq. 5. The same weighting functions of *w*_1_(*t*) to *w*_3_(*t*) were used as in the original papers [6, 7]. Finally, the parameter *V*_0_ can be obtained from *K*_1_ and *k*_2_ using Eq. 4 [6].

Details of the slice-by-slice calculation method are described elsewhere [6, 7, 22, 23]. In brief, a single delay time estimated from the mean TAC of each slice of the brain was used in the calculation program based on Eqs. 4 and 5 with fixed dispersion of 4 s (Fig. 1). A MATLAB toolkit included in the EMMA (Extensible MATLAB Medical image Analysis: http://www.bic.mni.mcgill.ca/software/emma/) programs developed at the Montreal Neurological Institute (McGill University, Montreal, Canada) was modified and applied for the calculation of the weighted integration method. In the pixel-by-pixel calculation, pixelwise correction for delay and dispersion of the arterial input function was applied to measurement of CBF and *V*_0_ (Fig. 1). The programs for the pixel-by-pixel were modified from the slice-by-slice calculation described above. An individual mask image to exclude radioactivity outside of the brain obtained from the average tissue activity image was applied to the dynamic PET data before CBF and *V*_0_ calculation to reduce the calculation time. The final parametric PET images were smoothed using a Gaussian filter with a size of 6 mm.

Cerebral vascular reactivity (CVR) was calculated as the percentage change in CBF from images before and after the ACZ challenge test for both slice-by-slice (CVR_s_) and pixel-by-pixel (CVR_p_) delay correction.

### Error analyses in CBF and *V*_0_ calculation

Error analyses were performed to investigate the biases of the CBF and *V*_0_ estimations with simulated delay time shifts. Several small ROIs 10-mm in diameter, including about 30 voxels, were drawn on the brain cortex of parametric images for various values of CBF (30–70 mL/min/100 g) and *V*_0_ (0.3–2.0 mL/100 g) to obtain tissue TACs from dynamic PET data. The arterial input function after the delay and dispersion correction for the brain tissue TAC (=*C*_a_) was shifted from −3 to 3 s in steps of 0.5 s in the analyses. Using these two TACs of tissue activity and shifted *C*_a_, CBF and *V*_0_ were recalculated based on a 1-TCM. Percent changes from the original CBF and *V*_0_ values were calculated and plotted to observe the effect of delay estimation errors on parametric values.

### Statistical analysis

In the MCA territory, 30 circular ROIs 10 mm in diameter were set for each hemisphere. The same ROIs were applied to all parametric images of individual subjects. For statistical analysis, the SPSS ver. 18 (IBM Co., Armonk, NY, USA) was used. Repeated measures analysis of variance (ANOVA) with a post-hoc test by the Student–Newman–Keuls method was applied to analyze the difference in estimated hemodynamic parameters between ipsilateral and contralateral sides, as well as CBF and *V*_0_ values before and after ACZ administration. CBF and *V*_0_ values from the different delay correction methods were also compared. *P* < 0.05 was considered to be statistically significant. Correlation analyses were conducted among different PET parameters from this study to observe relationships as well as to confirm theoretical changes in cerebral hemodynamics.

## Results

Representative TACs from arterial blood sampling with dispersion correction for the external tube (TAC_a_1), maximum ROI values in the ICA region (TAC_CA_), and one of the pixels in the brain tissue (TAC_bp_) from both high and low perfused regions are given in Fig. 2. After TAC_a_2 was estimated by delay and dispersion correction of TAC_a_1, the true input function at each pixel level (*C*_a_) was obtained by delay and dispersion correction of TAC_a_2. The result of curve fitting between *C*_a_ and TAC_bp_ for CBF and *V*_0_ calculation is also given in Fig. 2 (dotted lines).

There were no differences in physiological data before and after the ACZ injection except for the arterial O_2_ partial pressure (77.5 ± 9.4 vs. 86.5 ± 9.9 mmHg, *P* < 0.001). Mean values of hemodynamic parameters in the MCA region are given in Table 1. All parametric data showed significant differences between the two hemispheres; CBF, V_0_ and CMRO_2_ were significantly lower and CBV and OEF were significantly higher in the affected side compared with the contralateral hemisphere (*P* < 0.05). In the comparison between pixel-by-pixel and slice-by-slice delay correction methods, *V*_0_ values were significantly greater with the slice-by-slice than with the pixel-by-pixel correction method both before and after ACZ administration; however, CBF from the different correction methods did not show significant differences in both conditions. CVR values were significantly smaller in the ipsilateral side than the contralateral side for both pixel-by-pixel and slice-by-slice delay correction, and the two methods provided very close values. The delay image showed a significantly longer mean delay time in the affected hemisphere compared to that in the contralateral side (2.81 ± 0.14 s vs. 2.66 ± 0.15 s, *P* < 0.05). The asymmetry indices for the average delay times of the ipsi- to the contra-lateral hemispheres were in the range of 1.00–1.13.

**Table 1:** Hemodynamic parameters of ^15^O-PET study in patients

Parameter	Ipsilateral		Contralateral	
	Slice-by-slice	Pixel-by-pixel	Slice-by-slice	Pixel-by-pixel
CBF_B_ (mL/100 g /min)	45.1 ± 6.4^*^	46.5 ± 7.3^*^	50.6 ± 5.6	52.6 ± 6.8
CBF_A_ (mL/100 g /min)	52.8 ± 7.4^*$^	54.2 ± 8.6^*$^	63.1 ± 5.5^$^	65.7 ± 5.6^$^
Baseline *V*_0_ (mL/100 g)	2.54 ± 0.73^*†^	1.37 ± 0.64^*† ^	3.08 ± 1.03^†^	1.75 ± 0.72^† ^
After ACZ V_0_ (mL/100 g)	2.55 ± 0.87^*†^	1.39 ± 0.69^*†^	3.59 ± 0.76^†^	2.13 ± 1.07^†^
CVR (%)	17.4 ± 11.1^*^	17.0 ± 12.3^*^	25.5 ± 9.6	25.7 ± 10.5
Delay (s)	2.81 ± 0.14^*^		2.66 ± 0.15	
CMRO_2_ (mL/100 g/min)	2.87 ± 0.31^*^		3.06 ± 0.32	
OEF (%)	50.7 ± 4.8^*^		45.9 ± 5.7	
CBV (mL/100 g)	3.91 ± 0.70^*^		3.59 ± 0.72	

*ACZ* acetazolamide, *CBF*_B_ baseline cerebral blood flow, *CBF*_A_ cerebral blood flow after ACZ; *CMRO*_2_ cerebral metabolic rate of oxygen, *OEF* oxygen extraction fraction, *CBV* cerebral blood volume

^*^ *P* < 0.05 ipsi- vs. contra-lateral; ^†^ *P* < 0.05 slice-by-slice vs. pixel-by-pixel delay correction; ^$^ *P* < 0.05 before and after ACZ

**Fig. 3:**
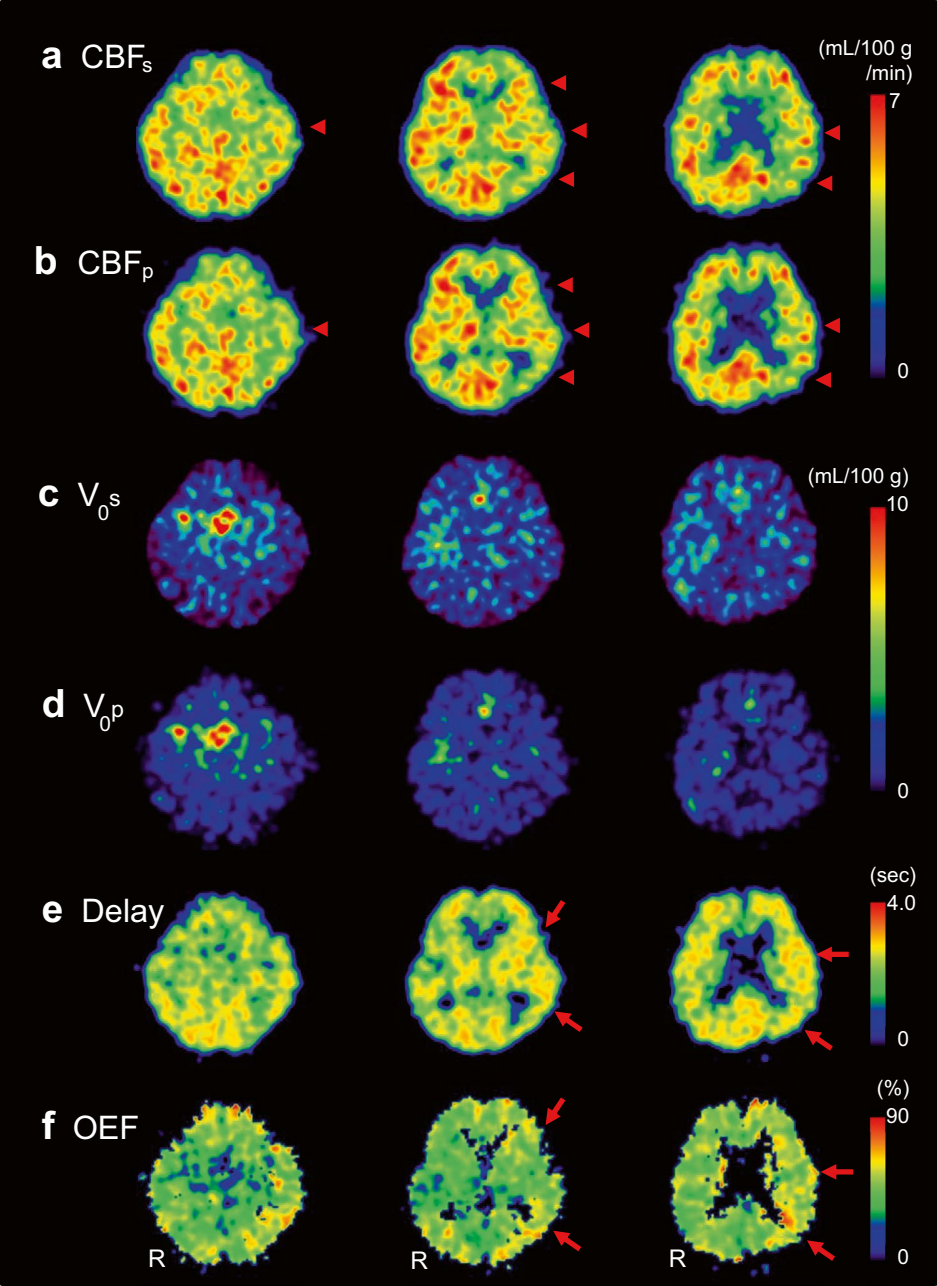
Images from a representative case with left ICA stenosis (R at the *bottom* of each column shows *right* side of the patient): Baseline CBF images calculated by slice-by-slice **a** and pixel-by-pixel **b** delay correction show a CBF decrease in left MCA territory. *Arrowheads* indicate CBF decrease in the affected side. CBF values were very close between the two methods. *Baseline V*_0_ images obtained by slice-by-slice **c** and pixel-by-pixel **d** delay correction show a significant difference in quantitative values between the two methods (2.08 vs. 1.27 mL/100 g in the global *V*_0_ mean). Asymmetry indexes (% difference between the hemispheres) were 78 and 72% for slice-by-slice and pixel-by-pixel methods, respectively. A delay image **e** from the pixel-by-pixel method shows a longer delay time in the left hemisphere in accordance with the OEF elevation (**f**) (*arrows*)

Parametric images of a representative case of left ICA stenosis are given in Fig. 3. CBF images with pixel-by-pixel delay correction (Fig. 3b) show similar cortical regional values to those from slice-by-slice delay correction (Fig. 3a). *V*_0_ images show significantly different quantitative values in the whole brain between the two delay correction methods (Fig. 3c, d). The delay image (Fig. 3e) shows the laterality of tracer arrival to be slightly longer in the affected left side (arrows) than in the contralateral side. The OEF image shows an ipsilateral increase compared with the contralateral side (Fig. 3f).

**Fig. 4:**
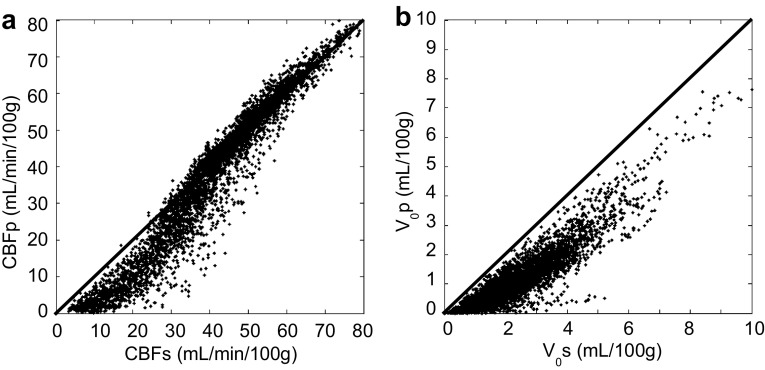
Pixelwise comparison of baseline CBF **a** and *V*_0_ **b** between the methods of slice-by-slice (CBFs and *V*_0_s) and pixel-by-pixel (CBFp and *V*_0_p) delay correction. *Scatter plots* were obtained from pixel values of a representative image slice

Figure 4 shows a pixelwise comparison between the two delay correction methods of pixel-by-pixel and slice-by-slice, in which CBF pixel values from the latter method were overestimated in the lower range although CBF values around 35 (mL/100 g/min) or higher showed a good correlation between the two methods. In contrast, *V*_0_ pixel values from the slice-by-slice delay correction method were overestimated in the whole brain compared with the pixel-by-pixel correction.

**Fig. 5:**
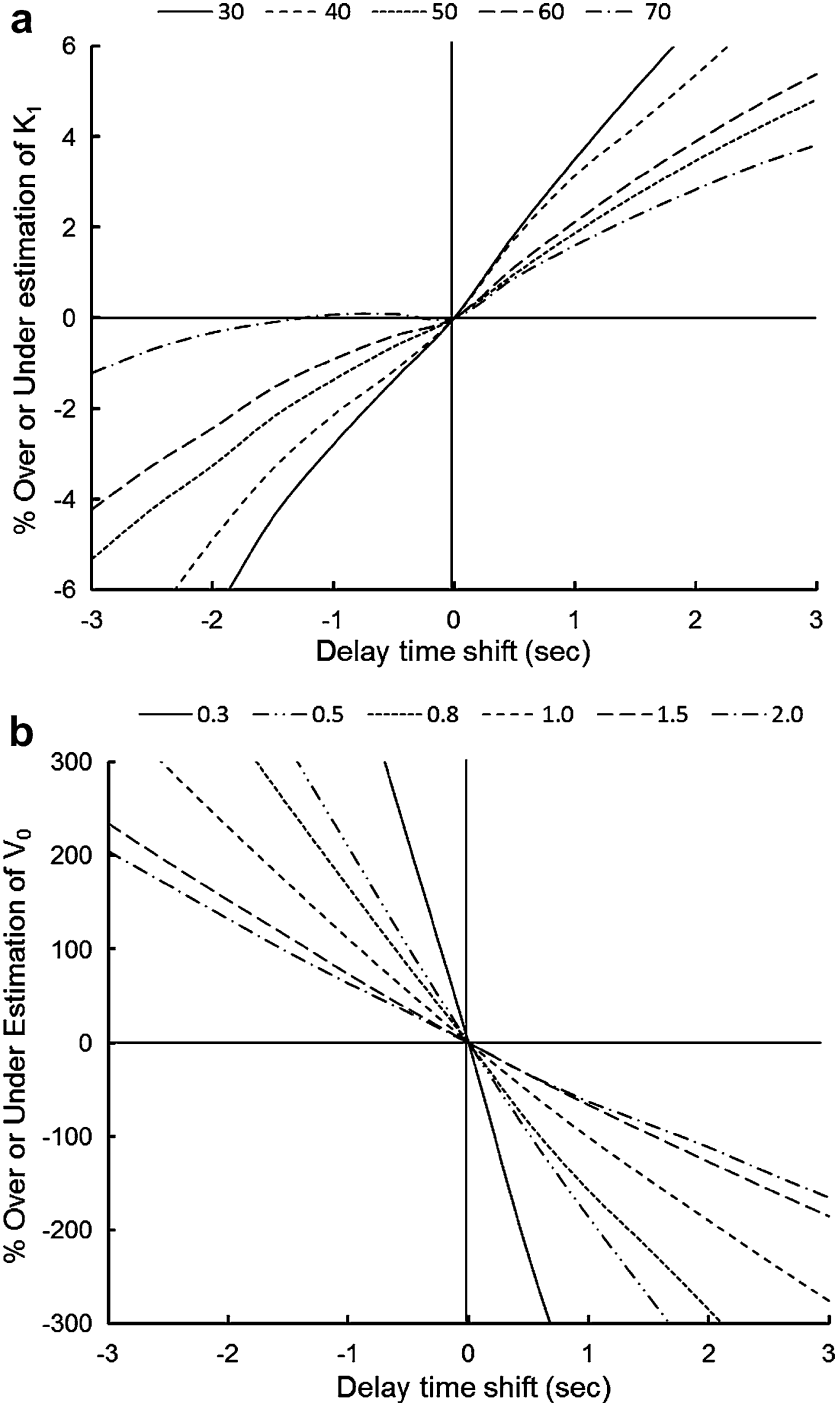
Results from error analyses assessed for effects of delay time shift on CBF **a** and *V*_0_ **b** values. A *negative* time shift assumes a delayed tracer arrival at the local brain region compared with the true input function, and a *positive* shift indicates an advanced tracer arrival. *Different lines* show various regional values of CBF (mL/min/100 g) and *V*_0_ (mL/100 g) as given at the *top* of the graphs

Figure 5 shows analyses of the effects of estimation error in the delay time correction on CBF and *V*_0_ values (time-shift simulation of the arterial input function from the true input function). A negative time shift indicates a delayed tracer arrival compared to the true input function at the local brain region, and a positive shift represents an advanced tracer arrival. Both parameters showed greater estimation errors in the longer time shift from the true input function; however, the error range in CBF was significantly smaller than the estimation errors observed in *V*_0_ calculation. Smaller parametric values tended to show greater errors in error estimation of delay time in both parameters.

**Fig. 6:**
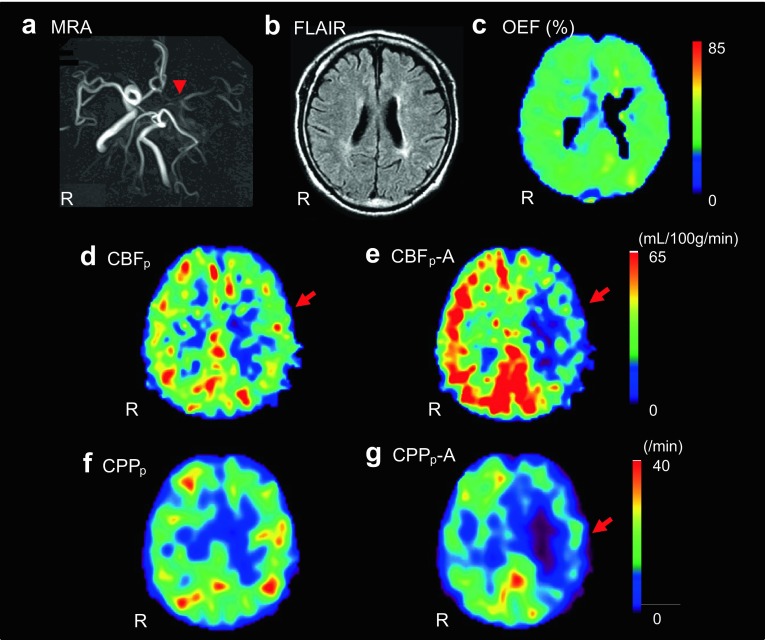
A representative case from this study. The patient had left ICA occlusion on MRA (**a**). *Arrowhead* shows the occlusion of ICA. MRI FLAIR image showed no significant ischemic region in the MCA territory (**b**). Although OEF image showed no significant increase in the left cerebral hemisphere (**c**), baseline CBF_p_ showed a tendency of decrease in the MCA territory (**d**). ACZ challenge test showed significant decrease in CBF_p_ (e) and CPP_p_ (**f** baseline, **g** after ACZ) in the affected region. The study indicated significant hemodynamic impairment because of the significant decrease in CVR and CPP, and the patient underwent EC–IC bypass surgery. CBF_p_-A and CPP_p_-A means pixel-by-pixel calculated CBF and CPP after ACZ. R on *each panel* represents *right side* of the patient. *Red arrows* show affected side of MCA territory

Figure 6 shows a representative case with left ICA occlusion, whose PET results significantly affected a treatment decision. Although the ^15^O-PET study showed a slight decrease in baseline CBF in the MCA territory (Fig. 6d), no significant OEF increase was observed in the affected region (Fig. 6c). The ACZ challenge test showed a significant decrease in CVR (Fig. 6e), but baseline CBF did not decrease significantly, and the results indicated stage I hemodynamic impairment. The cerebral perfusion pressure (CPP) image calculated from CBF/*V*_0_ showed a significant decrease after ACZ administration in left MCA territory (Fig. 6f, g), which indicated significant hemodynamic impairment. The patient underwent EC–IC bypass surgery because of the PET results and his symptoms of TIA.

**Fig. 7:**
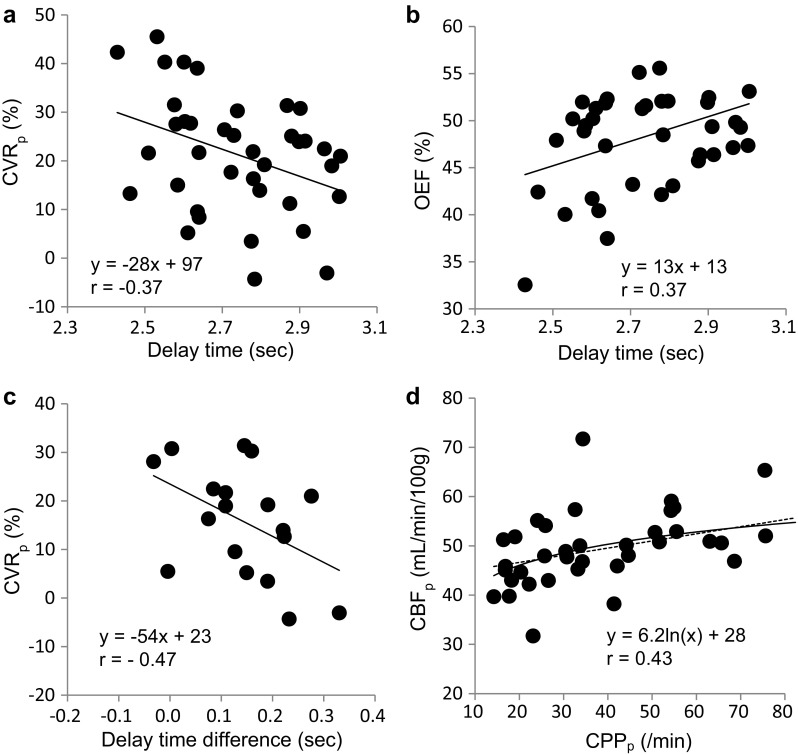
Different hemodynamic parameters were plotted from pixel-by-pixel delay estimation. The best fit regression line for correlation analysis was selected for each graph. **a** Delay time vs. CVR_p_, (*P* < 0.05), **b** Delay vs. OEF, (*P* < 0.05), **c** Delay time difference between contra- and ipsi-lateral hemisphere vs. CVR_p_, (*P* < 0.05), **d** arterial cerebral perfusion pressure (CPP_p_) obtained from CBF/V_0_p vs. CBF_p_ (*P* < 0.01). *Dashed line* shows linear regression (*y* = 0.15*x* + 43.7, *r* = 0.39, *P* < 0.05)

Figure 7 shows the relationships of hemodynamic parameters obtained from this study. Although CBF did not show a correlation with delay time, CVR_p_ and OEF had significant negative (a: *r* = −0.37) and positive (b: *r* = 0.37) correlations with delay time, respectively (*P* < 0.05). The difference of delay time between the contralateral to the ipsilateral hemisphere showed a significant correlation with CVR_p_ as well (c: *r* = − 0.47, *P* < 0.05). If we calculate cerebral perfusion pressure (CPP) with an equation of CBF/*V*_0_, CPP_p_ showed a good correlation with CBF linearly (*r* = 0.39, *P* < 0.05) or rather better in a logarithmic function (d: *r* = 0.43, *P* < 0.01). In contrast, *V*_0_ and CBV did not show a significant correlation (*r* = 0.24).

## Discussion

In the present study, a pixel-by-pixel delay time estimation for accurate correction was employed to calculate precise CBF and *V*_0_ images. The mean values of cortical CBF were not significantly different between the methods and the scatter plots showed a good correlation in the moderate to high perfusion range although the slice-by-slice correction method overestimated CBF values in the hypoperfused regions. Since the brain hypoperfused regions should have a longer delay time shift and errors were greater compared with the normal and high blood flow regions, CBF in those regions would have been overestimated in the slice-by-slice method (Figs. 5, 7). This result is important in a clinical setting because the hypoperfused regions are the target for hemodynamics evaluation and OEF increase due to CBF reduction should be the high risk region of hemodynamic infarction [24]. The ACZ challenge test with the perfusion SPECT or PET study is also useful for evaluating hemodynamic impairment in CVD patients, and it is used as an indication for EC–IC bypass surgery and other neurosurgical treatments in Japan [25, 26]. In both studies, precise measurement of CBF is very important.

*V*_0_ values, however, showed significant difference between the two methods in the whole *V*_0_ range of the brain because a slight shift of delay time estimation results in significant calculation errors [7]. The pixelwise delay for each brain location from TAC_a_2 was estimated in a narrow range of 0–4 s, which reduced the image calculation time compared with direct pixel-by-pixel delay estimation from the TAC_a_1, and provided an accurate delay time for *V*_0_ calculation. This delay of tracer arrival time from the ICA was almost identical to that in a previous study [9]. Direct delay estimation of TAC_bp_ from TAC_a_1 provided a greater range of delay between −5 s to more than 10 s depending on sclerotic and stenotic changes in the arteries of the blood sampling site and the local brain compared with our two-step correction method. The two-step method can avoid the effect of sclerotic changes in the brachial artery by adjusting TAC_a_1 to TAC_CA_ at first. Furthermore, the advantage of the pixel-by-pixel delay estimation is that it produces a delay time image which is a similar parameter to time-to-peak or arterial transit-time in MR perfusion studies, that would be beneficial information for the assessment of hemodynamic status in CVD patients. Because the delay time was correlated with OEF and CVR (Fig. 7), it may be able to estimate OEF elevation and CVR reduction without a ^15^O_2_ scan or the ACZ challenge test. If the image derived input function method with ^15^O-water PET is established, the delay image might be beneficial as one of the screening tools.

Previous studies reported that CBF values are sensitive to errors in delay time estimation, where a positive delay time shift shows overestimation and a negative shift underestimates CBF values [7, 9, 27]. We also observed similar estimation errors from the error analyses (Fig. 5a). The percent change in CBF values of less than 7% in the range of − 2 to 2 s shift, which is consistent with the report by Iida et al. [9], seems small enough and acceptable. In the present study, the difference of CBF values between slice-by-slice and pixel-by-pixel delay correction was less than 4% in the cortical region, and the difference was not significant in statistical analysis. However, significantly longer delay times in the severely affected brain regions may induce significant errors in the slice-by-slice correction. Our two-step method with the positive delay time shift (0–4 s) seems more robust in CBF measurement with negligible errors compared to the slice-by-slice direct correction method.

**Fig. 8:**
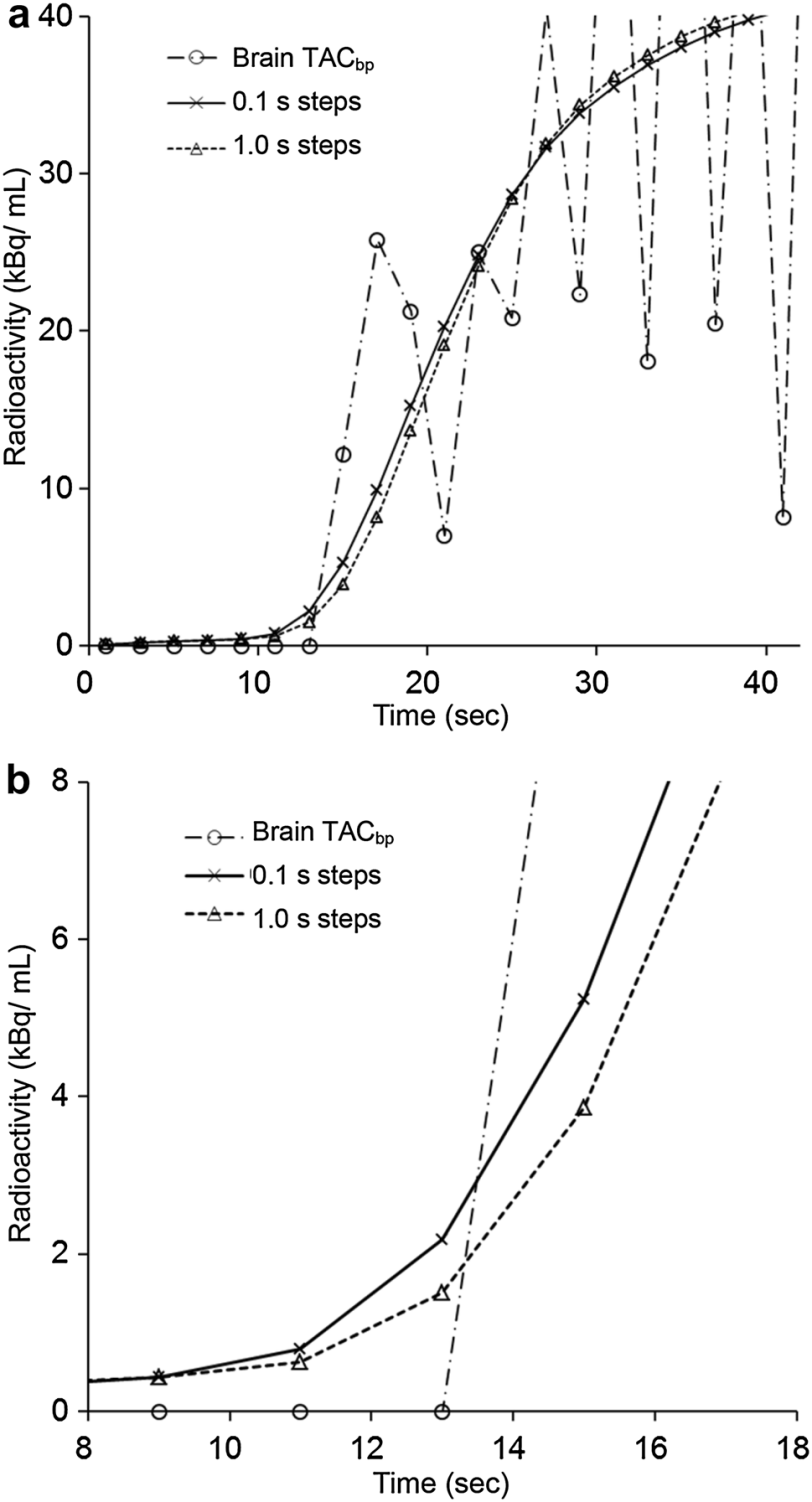
Representative curves of TAC_bp_ and TAC_a_2 after fitting using the least-squares method for delay estimation (**a**) and zoom of the graph between 8 and 18 s (**b**). 0.1 s steps showed a better fitting curve (*solid line*) than 1.0 s steps (*dotted line*) and delay times from these fittings were estimated to be 1.6 and 2.0 s for 0.1 and 1.0 s steps, respectively. These delay times estimated quantitative values of this voxel to be 34.1 and 33.5 (mL/100 g/min) for CBF and 0.9 and 1.4 (mL/100 g) for *V*_0_, respectively. The statistical analysis showed that CBF_p_ was not different in cortical values, but V_0_p was significantly different for both hemispheres

In contrast, *V*_0_ values seem to be more vulnerable to errors in delay estimation than in CBF [7]. The error range is not negligible because only a 1 s time shift may cause 50–200% difference in the *V*_0_ range of 0.5–2.0 (mL/100 g) (Fig. 5b). The significant overestimation of *V*_0_ values with a small error of delay estimation in the slice-by-slice method may have a severe effect in a clinical setting, which confirms the necessity of pixel-by-pixel delay correction. The *V*_0_ apparently reflects the arterial-to-capillary blood volume, which is different from CBV including venous volume [7, 11, 28]. To evaluate cerebral perfusion pressure (CPP) from PET parameters, CBF/*V*_0_ would be more appropriate than CBF/CBV values [14, 28], and images with precise regional values of *V*_0_ offer beneficial information for the assessment of hemodynamics of CVD. The high temporal resolution (0.1 s) for delay time estimation between TAC_a_2 and C_bp_ in the present study is preferable for this precise estimation of PET parameters because different time steps of 0.1 and 1.0 s for delay estimation showed differences in delay time as well as *V*_0_ values (Fig. 8). The residuals for the fittings with 1.0, 0.1 and 0.01 s steps were almost the same and CBF values did not show differences; however, the 1.0 s step delay time estimation provided significantly different *V*_0_ values not only in the affected side, but also in the contralateral side compared with those of 0.1 or 0.01 s steps (data not shown). Because the different time steps of 0.1 and 0.01 s did not lead to differences in the results of delay, CBF and *V*_0_, we decided to use 0.1 s steps for delay time estimation. The calculation time of the delay estimation was not much affected by difference in the temporal resolution (step size). However, least-squares fitting for CBF and *V*_0_ calculation using Eq. 3 is still time consuming even after recent improvements in computer power. The weighted integration method reduces calculation time significantly as previously reported [6, 7, 29].

Regional CVR_p_, which reflects the vasodilatory capacity, showed a significant correlation with delay time and asymmetry of delay (Fig. 7a, c). The negative correlation represents a longer tracer arrival time in the severely affected region, which is consistent with impaired hemodynamic change in the area. Although the means of delay time in the ipsi- and contra-lateral hemispheres showed only 0.15 s difference (Table 1), a mean value is often blurred by the mixing of significant and insignificant results, and the statistical analysis showed that the difference was significant. Parameters of oxygen metabolism measured by ^15^O-gas PET could be compared with the new parameters in the present study because this is a retrospective study to propose the better analyses of patients’ hemodynamic conditions using ^15^O-water PET. OEF showed a significant correlation with delay time (Fig. 7b), indicating that delay time is closely correlated with hemodynamic impairment. The lower range of CPP showed a positive correlation with CBF although CBF tended to reach a plateau value in higher CPP. This correlation is also consistent with the theory of hemodynamic changes [14, 15, 30], as we previously reported the importance of precise estimation of V_0_ for the CPP evaluation [28].

In the present study, the pixel-by-pixel calculation estimated *V*_0_ in the contralateral hemisphere to be about 49% of CBV, which is close to the values in the previous reports (about 20–40%) [31, 32]. However, the slice-by-slice delay correction estimated contralateral *V*_0_ to be about 87% of CBV, which is greater than our previous studies showing *V*_0_ values of less than 60% of CBV [11, 28]. Although the difference in the initial frame time (5 s in the previous studies) may have provided greater *V*_0_ values in the slice-by-slice calculation method, the initial slope at tracer arrival was estimated better in 2-sec than in 5-s frames, especially when obtaining the TAC_CA_. The coefficient of variation (COV) values were calculated from ROIs drawn on each cerebral hemisphere of the patients. CBF images showed no difference in COV (0.18 ± 0.02 for both calculation methods), and *V*_0_ images showed 0.37 ± 0.07 and 0.50 ± 0.11 for the slice-by-slice and pixel-by-pixel methods, respectively. This result indicates that the *V*_0_ image was noisier in the latter method than in the former one, and improvement of image quality may be needed for *V*_0_ evaluation.

Since the patients enrolled in this study had only minor symptoms and half of them were asymptomatic, we might have seen different results from severely affected cerebral circulation in symptomatic patients. However, a tendency of hemodynamic impairment in the chronic phase of CVD was observed in the present study, suggesting that this method for precise estimation of delay time correction would be ideal for the assessment of CVD caused by stenoocclusive lesions in the major cerebral arteries. Baseline *V*_0_ tended to show smaller values in the ipsilateral side compared with the contralateral side (Table 1), and the result was similar to the previous works [28, 33]. In the chronic phase of CVD, the arteriole-to-capillary part of vessels may not necessarily show vasodilatory change, and increase in CBV is assumed to be caused mainly by increase in venous volume. In the present study, only a few cases were in the stage of misery perfusion assessed by a significant OEF increase, and these patients also did not show significant increase in ipsilateral *V*_0_. Microvascular impairment in cerebral ischemic change may cause capillary collapse in the chronic phase of CVD [34].

## Conclusion

The precise estimation of hemodynamic parameters such as CBF and *V*_0_ using the new method should be ideal and beneficial for clinical use. Pixel-by-pixel delay estimation also provides a new image of delay time that should provide useful information for the clinical assessment of CVD.
